# Clinical variables influencing the perception of fatigue in people with multiple sclerosis: a cross-sectional study using FSIQ-RMS

**DOI:** 10.1186/s12883-024-03643-x

**Published:** 2024-04-25

**Authors:** Giovanni Sellitto, Ilaria Ruotolo, Antonio Ianniello, Federica Felicetti, Giorgia D’Ambrosi, Anna Berardi, Giovanni Galeoto, Antonella Conte, Carlo Pozzilli

**Affiliations:** 1 MS Center, S. Andrea Hospital, Sapienza University, Rome, Italy; 2 IRCCS Neuromed, Pozzilli, Italy; 3 Department of Human Neurosciences, Sapienza University, Rome, Italy

**Keywords:** Multiple sclerosis, Fatigue, FSIQ-RMS, Disability

## Abstract

**Background:**

Physical fatigue is one of the most disabling symptoms in people with Multiple Sclerosis (PwMS). Several factors might influence the development of fatigue, such as gender, education, body mass index (BMI), Expanded Disability Status Scale (EDSS), disease duration, working status (Ws), physiotherapy (Ph), and disease-modifying therapies (DMTs). Fatigue Symptoms and Impacts Questionnaire-Relapsing Multiple Sclerosis (FSIQ-RMS) is a patient-reported outcome (PRO) that allows one to define the impact of fatigue in PwMS clearly. This study aimed to assess fatigue impact on PwMS by using FSIQ-RMS.

**Methods:**

The participants were enrolled from May to July 2021 in MS Centers of Sant’Andrea Hospital and Policlinico Umberto I Hospital in Rome. Fatigue was evaluated using the FSIQ-RMS, validated, and culturally adapted in Italian. Clinical and demographic data were collected at the same time.

**Results:**

We enrolled 178 PwMS [Female 74.16%; RMS 82.58%, SPMS 17.52%]. FSIQ-RMS scores were significantly correlated with EDSS (p-value < 0.01). Analysis of variance between means showed a statistically significant difference between the BMI groups at the 24hours_FSIQ-RMS score and the 7days_FSIQ-RMS score (*p* < 0.01), with the lower BMI group having the highest scores. Furthermore, perceived fatigue significantly improved both in subjects performing Ph (*p* < 0.05) and in those who actively work (*p* < 0.01).

**Conclusions:**

The use of FSIQ-RMS in a real-world setting confirmed that underweight and high levels of disability are closely related to fatigue. In addition, Ph and active Ws are strongly correlated with fatigue in PwMS.

## Background

Fatigue is perhaps the most common complaint associated with Multiple Sclerosis (MS), with prevalence estimates ranging between 70 and 90% [1, 2]. Fatigue impacts the social and working lives of people with MS (PwMS), representing one of the main factors related to the health-related quality of life (HRQoL) and a common reason for early retirement [3, 4]. Fatigue increases with age, and is more frequent in progressive MS [5]; however, its presence in the early stage of the disease, even at the time of the first episode suggestive of MS, is predictive of future disability [6, 7].

Given the subjective nature of fatigue, it is best evaluated via a patient-reported outcome (PRO) instrument. Although several available PRO instruments have been used so far in PwMS [8, 9], a review of their measurement properties suggests shortcomings in terms of current standards for PRO instrument development [10, 11]. Recently, to address limitations of existing MS-specific instruments, a new content-valid, concise PRO instrument to assess fatigue symptoms relevant to patients within the spectrum of MS, and in accordance with the Food and Drugs Administration (FDA) PRO guidance, has been developed. The instrument named the “Fatigue Symptoms and Impacts Questionnaire-Relapsing Multiple Sclerosis” (FSIQ-RMS) [12] has been proven to be a reliable PRO instrument that has demonstrated content and measurement validity for fatigue-related symptom and impact items. FSIQ-RMS examines two areas of fatigue’s physical, cognitive, and emotional impact. The first concerns the fatigue experienced in the previous 24 h. The second includes items that investigate the impact of fatigue in the previous 7 days. Its responsiveness and meaningful change of FSIQ-RMS have been analyzed in one international phase III trial, the Optimum Trial, which represents the first study to implement a validated disease-specific fatigue measure (PRO) as a prespecified endpoint [13]. Recently, FSIQ-RMS has been translated and validated for the Italian population [14]. The Italian version of the FSIQ-RMS showed excellent internal consistency (Cronbach’s alpha 0.92) and test-retest reliability (ICC 0.96) for both domains.

With the increasing numbers of multinational and multicultural studies, adequate translation, cross-cultural adaptation and psychometric testing are crucial when introducing questionnaires in new countries. Cross-cultural adaptation is a process that looks at both translational and cultural issues when a questionnaire is being prepared for use in another country [15].

Translation in Italian of the FSIQ-RMS was performed by PharmaQuest Group but, as far as we know, the assessment of the usefulness and intercultural adaptation has not yet been carried out. Thus, the aim of the study was to culturally adapt the Italian version of FSIQ-RMS.

## Methods

PwMS were enrolled from May to July 2021 in two Italian MS Centers, S. Andrea Hospital and Policlinico Umberto I Hospital in Rome. The enrolment of the participants was carried out during the study of the Italian translation and validation of the FSIQ-RMS scale.

The study was approved by the Ethics Committee of the participating hospitals and was performed in accordance with the 1964 Declaration of Helsinki and its later amendments.

The inclusion criteria were: diagnosis of MS [both relapsing MS and progressive (PMS)], according to the 2017 McDonald’s criteria [16], age between 18 and 70 years and ability to participate in a 90-min face-to-face interview, medical record available for the previous 12 months or from the time of RMS diagnosis, and being fluent in Italian. Patients with the following characteristics were excluded: any condition that may cause energy-related or fatigue-related symptoms (different from MS); ongoing treatment for an autoimmune disease other than MS; history of suicide attempts and concomitant participation in trials with experimental drugs for any condition. Patients deemed by the treating neurologist to have clinically significant cognitive impairment were also excluded, as they may not have been able to comply with the study procedures [17]. .

Study subjects had to complete the FSIQ-RMS scale. This test aims to measure the level of perceived fatigue in our sample of PwMS, using its two components at 24 h (24hours_FSIQ-RMS) and 7days (7days_FSIQ-RMS). The former is the fatigue domain and includes 7 items regarding fatigue experienced in the preceding 24 h. The latter is the impact domain which encompasses 13 items assessing the physical, cognitive, emotional, and coping impacts of fatigue over the previous 7 days. The higher each score, the greater the perception and the impact of fatigue.

The total scores obtained at the 24hours_FSIQ-RMS and the 7days_FSIQ-RMS were then correlated independently with the clinical and demographic variables detected in our study group.

The variables potentially correlated with fatigue were gender, education, Body Mass Index (BMI), Expanded Disability Status Scale (EDSS) [18], disease duration, working status (Ws), physiotherapy (Ph), and disease-modifying therapies (DMTs).

Some of these variables were subdivided into categories. The demographic variables that we decided to categorize are education, according to the five levels of the Italian school system, and the BMI, creating four groups, as defined by the international guidelines [19].

Regarding the clinical variables, we divided treatment with disease-modifying treatments (DMTs) into three categories. Based on the efficacy of the drugs, we distinguish the Moderate Efficacy Therapies (METs) and High Efficacy Therapies (HETs), adding a third category for currently untreated patients (W_DMTS) [20].

The software used for the statistical analyses was SPSS Statistics version 25. To compare categories, a Student’s t-test or analysis of variance (ANOVA) was performed [21]. Independent Student’s t-test was used to compare dichotomized variables, given a sample size > 30 observations; while One-way ANOVA was used for categorized variables involving more than 2 independent groups. Parametric tests are based on assumptions about the distribution of the underlying population from which the sample was taken. The hypothesis is that, given the sample size exceeding 30 observations, the data are distributed approximately normally [22].

Finally, Pearson’s linear correlation analysis was carried out to correlate the continuous variables and the FSIQ-RMS scores [23]. Data were expressed as mean ± SD.

## Results

We enrolled 178 PwMS [Female 74.16%; RMS 82.58%, SPMS 17.52%] aged 47.38 ± 12.34 years, with a median EDSS score of 3.07 ± 1.81 SD. The disease duration was 12.6 ± 9.5years.

Clinical and demographic findings are shown in Table 1.

**Table 1:** Clinical and demographic characteristics of the study sample. *sd = Standard Deviation; EDSS = Expanded Disability Status Scale; METs = Moderate Efficacy Therapies; HETs = High Efficacy Therapies; W = Wtihout; RMS = Relapsing Multiple Sclerosis; SPMS = Secondary Progressive Multiple Sclerosis

Age, years, mean ± sd*	47.28 ± 12.34
Gender, *n* (%)	
Female	132 (74.16)
**Education, ** *n* ** (%)**	
Elementary school	2 (1.12)
Middle school	28 (15.73)
High school	84 (47.19)
Three-year degree	12 (6.74)
Master’s degree	52 (29.21)
**Body Mass Index (BMI), ** *n * **(%)**	
Underweight	11 (6.18)
Healthy	109 (61.24)
Overweight	47 (24.40)
Obese	11 (6.18)
EDSS*, mean ± sd*	3.07 ± 1.81
Duration of illness, mean ± sd*	12.58 ± 9.50
**Disease modyfing therapies (DTMs), ** *n * **(%)**	
METs*	78 (47.56)
HETs*	34 (20.73)
W*_DMTs	52 (31.71)
**Physiotherapy training during study, ** *n* ** (%)**	
No	132 (74.14)
**Working status during study, ** *n * **(%)**	
No	62 (34.83)
**Clinical phenotypes, ** *n * **(%)**	
RMS*	147 (82.58)
SPMS*	31 (17.52)

The ANOVA analysis performed on the categorical variables gender, education and DMTs did not show any statistical difference between the groups in the 24hours_FSIQ-RMS score and the 7days_FSIQ-RMS score

**Fig. 1:**
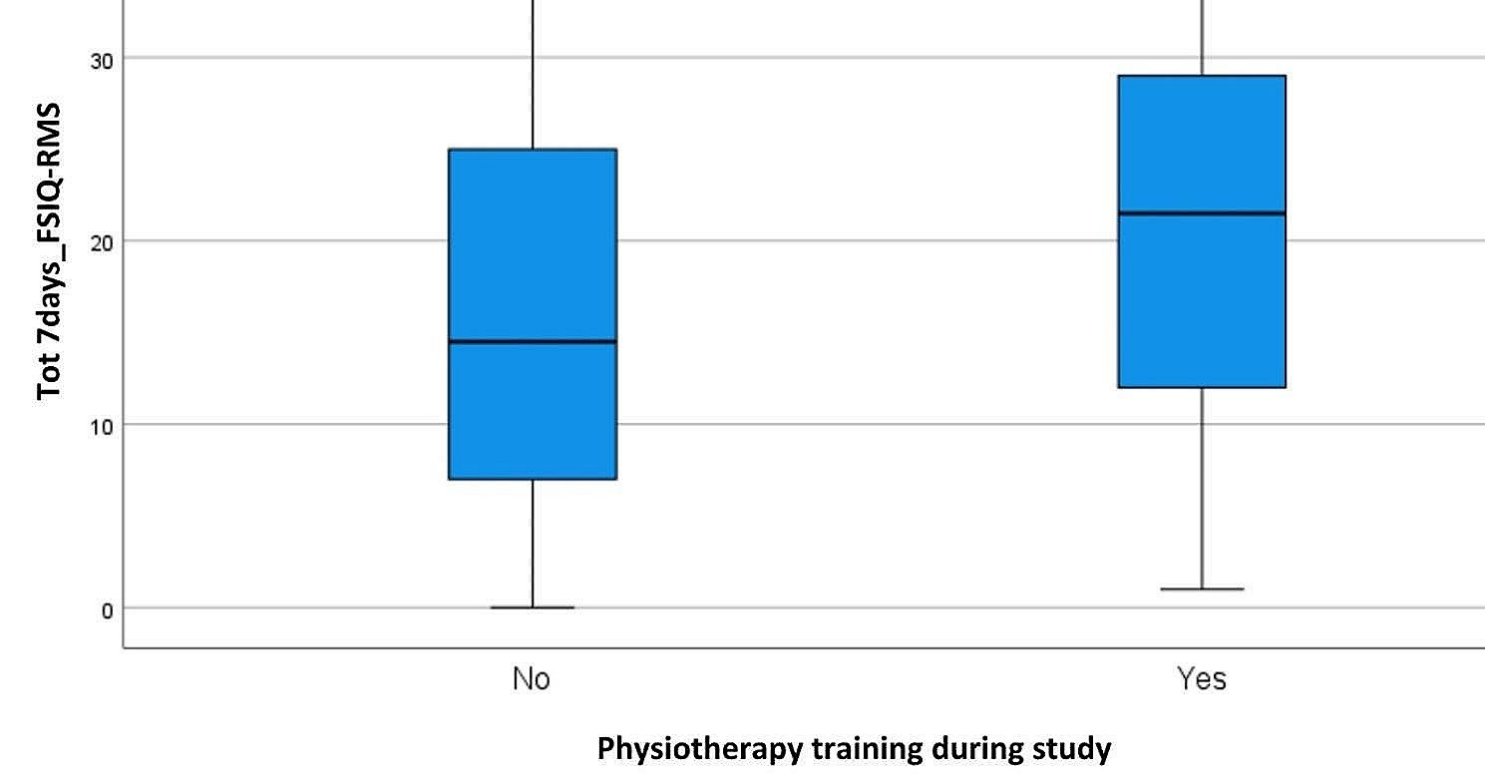
Box plot for physiotherapy training during study (Ph) at 7days_FSIQ-RMS

Regarding Ph, the differences between the two groups were not statistically significant in the 24hours_FSIQ-RMS score (*p* = 0.281). However, 7days_FSIQ-RMS score was significantly lower in the group performing Ph (*p* = 0.016) compared to the group not undergoing Ph, as shown in Fig. 1.

**Fig. 2:**
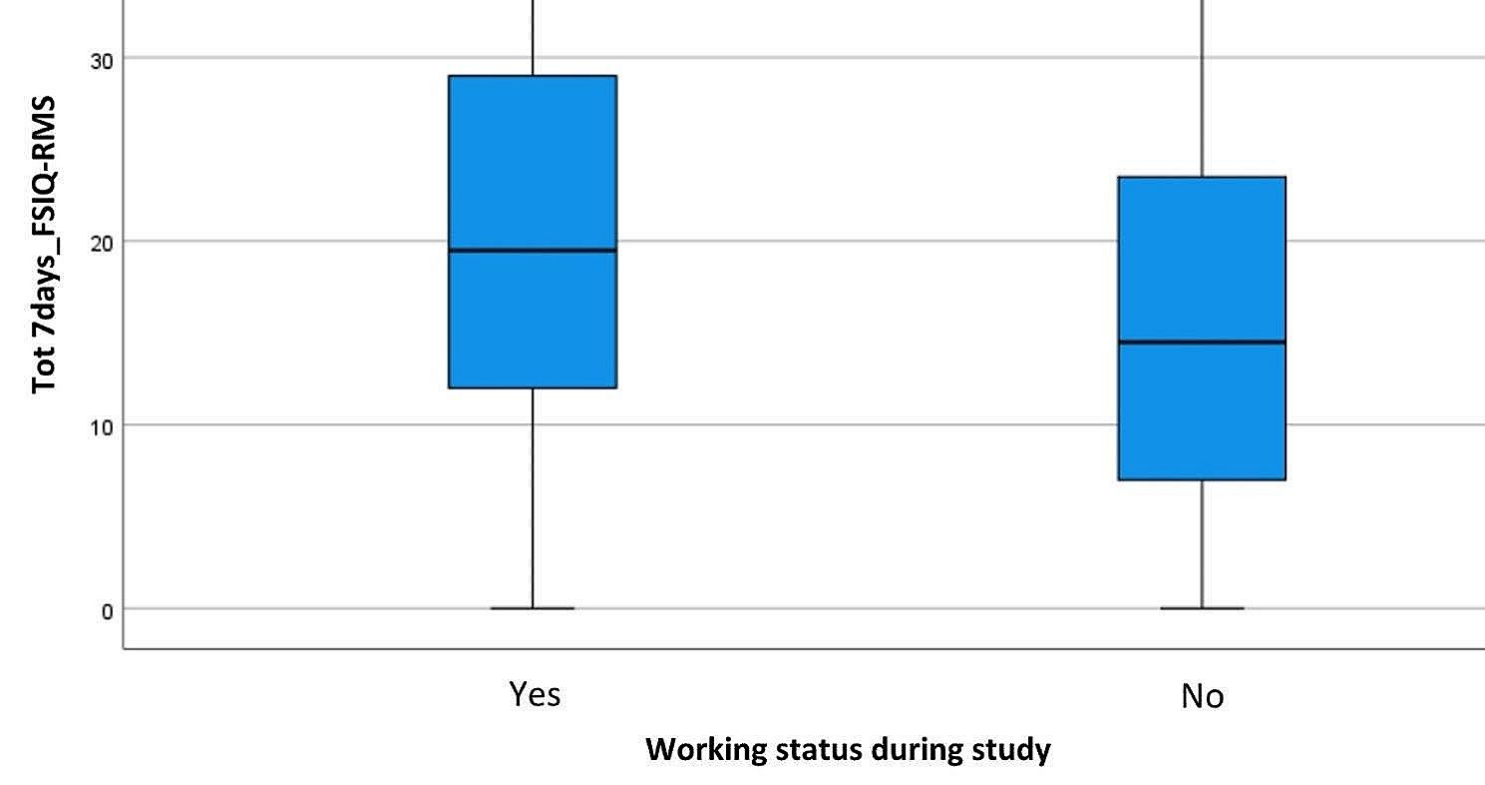
Box plot for working status during study (Ws) at 7days_FSIQ-RMS

**Fig. 3:**
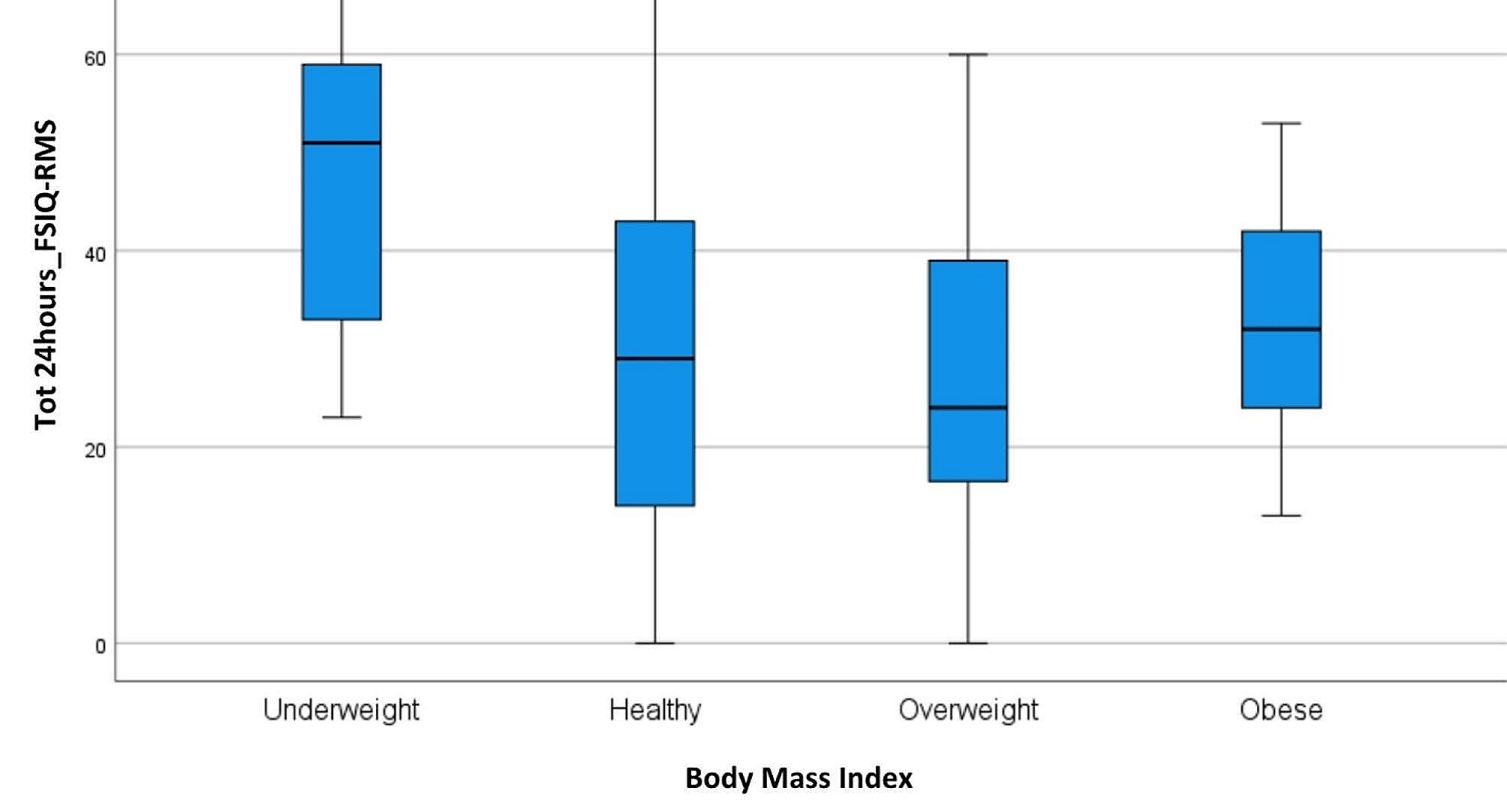
Box plot for body mass index (BMI) at 24hours_FSIQ-RMS

**Fig. 4:**
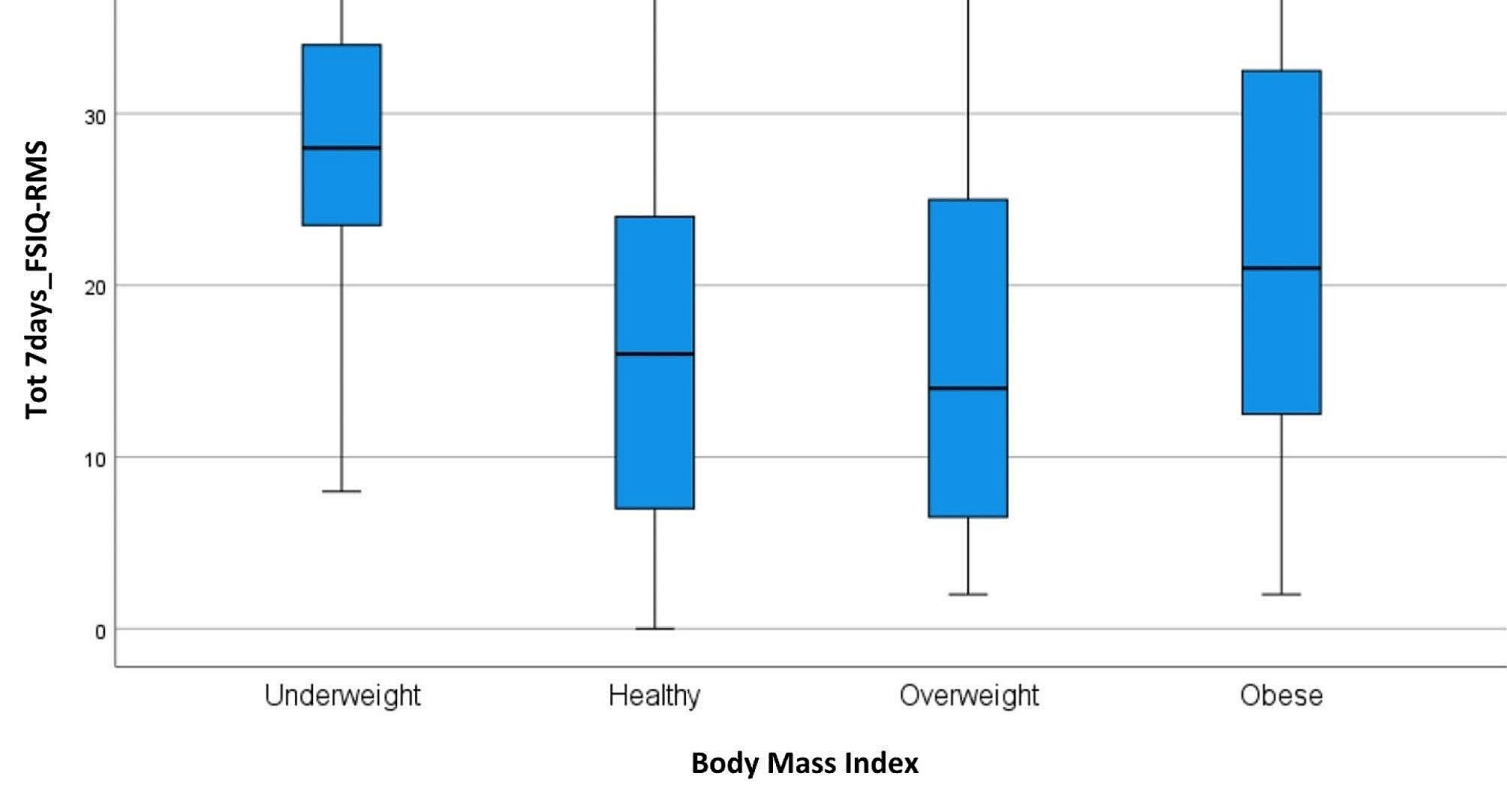
Box plot for body mass index (BMI) at 7days_FSIQ-RMS

A similar remark regards Ws. There were no significant differences between the two groups in the 24hours_FSIQ-RMS score, while the groups differed significantly in the 7days_FSIQ-RMS score (*p* < 0.01), graphically shown by Fig. 2. Interestingly, the underweight patient group reported significantly higher levels of fatigue than the others, both at 24 h (Fig. 3) and at 7days_FSIQ-RMS scores (Fig. 4), *p* < 0.01 in both cases. Details of the analysis of variance between the means are given in Table 2.

**Table 2:** Student t-test and analysis of variance (ANOVA) between averages for categoric variables

	24hours_FSIQ-RMS Mean, standard deviation	*P* value	7days_FSIQ-RMS Mean, standard deviation	*P* value
Gender				
Female	29.58 (± 17.190)	0.576	17.16 (± 10.546)	0.454
Male	30.24 (± 18.462)		16.72 (± 11.224)	
Body Mass Index (BMI)				
Underweight	47.73 (± 16.341)	0.003**	27.45 (± 8.501)	0.002**
Healthy	28.72 (± 17.676)		16.14 (± 10.185)	
Overweight	27.21 (± 16.130)		15.64 (± 10.631)	
Obese	32.82 (± 12.679)		21.64 (± 12.307)	
Education				
Elementary school	42,50 (± 10.607)	0.632	15,50 (± 3.536)	0.213
Middle school	31,79 (± 17.931)		20,39 (± 11.755	
High school	29,07 (± 17.287)		17,01 (± 10.962)	
Three-year degree	24,83 (± 12.583)		11,75 (± 6.662)	
Master’s degree	30,40 (± 18.751)		16,58 (± 10.327)	
Disease modyfing therapies (DTMs)				
METs	29,08 (± 17.381)	0.601	15,77 (± 9.840)	0.333
HETs	34,65 (± 19.289)		19,68 (± 11.438)	
W_DMTs	27,00 (± 17.016)		17,19 (± 11.287)	
Physiotherapy training during study				
Yes	32.15 (± 17.229)	0.281	20.30 (± 10.458)	0.016*
No	28.92 (± 17.550)		15.92 (± 10.580)	
Working status during study				
Yes	29.33 (± 18.243)	0.658	15.45 (10.414)	0.006**
No	30.55 (± 16.058)		20.03 (10.654)	

24h_FSIQ-RMS = 24 h total score Fatigue severity impact scale-relapsing Multiple Sclerosis; 7d_FSIQ-RMS = 7 days total score fatigue severity impact scale-relapsing Multiple Sclerosis; METs = Moderate Efficacy therapies; HETs = High Efficacy therapies; W_DMTs = Without_Disease modyfing therapies

*Statistical significance for *p* < 0.05

**Statistical significance for *p* < 0.01

Pearson’s linear correlation analysis applied to continuous variables revealed a direct correlation between fatigue and the EDSS regarding the 24hours_FSIQ_RMS score with r value (r) = 0.213 (*p* < 0.01). The correlation was even greater for the 7days_FSIQ-RMS score (*r* = 0.417, *p* < 0.001) (Table 3).

**Table 3:** Correlation between fatigue and continuous variables

		EDSS score		Duration of illness		
24h_FSIQ-RMS	Pearson Correlation	0,213		0,023		
	Sig. (2-tailed)	0,004**		0,758		
	*N*	178		178		
7d_FSIQ-RMS	Pearson Correlation	0,417		0,138		
	Sig. (2-tailed)	0,000*		0,067		
	*N*	178		178		

24h_FSIQ-RMS = 24 h total score Fatigue severity impact scale-relapsing Multiple Sclerosis; 7d_FSIQ-RMS= 7 days total score fatigue severity impact scale-relapsing Multiple Sclerosis; BMI = body mass index; EDSS = expanded disability status scale

*Correlation statistically significant for *p* < 0.001

*Correlation statistically significant for *p* < 0.01

Finally, we did not find significant correlations between fatigue and disease duration in our sample, considering neither the previous 24 h nor the previous 7 days.

## Discussion

Cross-cultural adaptation of the FSIQ-RMS instrument on a sample of the Italian MS population showed that physical, cognitive, emotional, and coping impacts of fatigue are influenced by physiotherapy and work commitment, whereas BMI and disability affect the perception of fatigue experienced.

A higher level of fatigue was observed in more disabled and underweight patients. On the other hand, physiotherapy as well as a working state were related to a reduction of fatigue in PwMS.

Several previous studies focused on this topic using different PRO instruments. The relationship between fatigue and disability measured according to the EDSS score has been well-known for years [5, 24]. More recently, looking at the predictors of patients who reported fatigue symptoms in a large Swedish nationwide register-based MS cohort, Englund S et al. 2022 [25] showed that among MS-associated characteristics, higher disability level stood as the best predictor of fatigue. This was not an unexpected finding since brain atrophy in specific brain areas is related to MS fatigue and an increased EDSS score [26, 27].

Of particular interest is the correlation that we found between BMI and 24hours_FSIQ-RMS, which assesses each subject’s individual experience of fatigue, based on their BMI. In our sample, this correlation was not observed in overweight or obese subjects but only in underweight people, indicating a higher level of fatigue experienced by underweight PwMS. This correlation comes with a caveat: BMI values were not homogeneously distributed among our subjects, and only 6.18% were underweight. Recent studies showed that an interventional diet regime significantly reduces perceived fatigue and BMI. However, the significant reduction in perceived fatigue appears to be independent of changes in markers of metabolic health [28–30]. More studies are needed to investigate this specific issue.

Our work also aimed at analyzing how the adapted FSIQ-RMS tool could detect the extent to which the fatigue construct contributes to differences between individuals who underwent physiotherapy and those who did not. We found a statistically significant difference between the group attending Ph and the one that did not, in terms of perceived fatigue (7days_FSIQ-RMS). This finding is widely supported by systematic reviews supporting a beneficial contribution to fatigue through different types of interventions [31–33]. A variety of physical therapy techniques, including endurance exercise programs, resistance exercise programs [34, 35], vigorous cool room treadmill training [36], specific balance exercises [37] and intensive circuit class therapy [24] provided to be effective in alleviating MS fatigue.

Regarding working status, our work confirmed higher levels of perceived fatigue experienced among the unemployed. As already known from the literature on this topic, PwMS are more likely to be unemployed or limited in fitness to work [38]. Moreover, fatigue is often considered the single most disabling symptom of MS and may be the main driver for loss of employment. On the other hand, loss of occupation may also lead to greater physical inactivity and can cause depression, further influencing the perception of fatigue [39].

We did not find any correlations between fatigue and other variables such as gender, education level, disease duration, and use of DMTs. Despite previous data on these parameters being conflicting [10, 40] we have to consider that disease duration is not homogeneous among studies (i.e., disease duration was shorter in our sample). Furthermore, the heterogeneity of the DMTs used by our sample might considerably affect the final results.

## Conclusion

As reported in our previous study, the FSIQ-RMS scale has proved to be a very reliable outcome measure of fatigue in MS. Furthermore, the assessment of three domains (physical impact, cognitive/emotional impact, and coping impact) as well as the simultaneous 24-hour and 7-day testing, allow for a more accurate QoL assessment [11]. The cultural adaptation of FSIQ-RMS in Italian PwMS shows the great efficacy of this instrument in capturing the positive effects of physiotherapy and employment status on the impacts of fatigue, as well as the significance of a lower disability status and maintaining a normal weight.

## Data Availability

The datasets used and/or analyzed during the current study are available from the corresponding author on reasonable request.

## Abbreviations

PwMS: People with Multiple Sclerosis
BMI: Body Mass Index
EDSS: Expanded Disability Status Scale
Ws: working status
Ph: physiotherapy
DMTs: disease modifying therapies
FSIQ-RMS: Fatigue Symptoms and Impacts Questionnaire-Relapsing Multiple Sclerosis
PRO: Patient-Reported Outcome
MS: Multiple Sclerosis
HRQoL: health-related quality of life
FDA: Food and Drugs Administration
METs: Moderate Efficacy Therapies
HETs: High Efficacy Therapies
